# Dietl Syndrome Misattributed to Gastrointestinal Disease in a Child: A Pediatric Diagnostic Pitfall

**DOI:** 10.7759/cureus.107480

**Published:** 2026-04-21

**Authors:** Rana Khazendar, Micah Pippin, Sanjay Shrestha

**Affiliations:** 1 Family Medicine, Louisiana State University Health Sciences Center, Alexandria, USA; 2 Family Medicine, Rapides Regional Medical Center, Alexandria, USA

**Keywords:** abdominal pain in children, dietl crisis, dietl syndrome, unilateral nephrectomy, ureteropelvic junction obstruction

## Abstract

Dietl syndrome is a rare manifestation of intermittent ureteropelvic junction (UPJ) obstruction characterized by episodic abdominal pain, nausea, and vomiting. In pediatric patients, symptoms are frequently misattributed to gastrointestinal or functional disorders, resulting in delayed diagnosis and potential irreversible renal injury. Diagnostic challenges can result in advanced disease before the underlying obstruction is recognized. Although gastrointestinal etiologies, such as constipation and functional abdominal pain, are common and reasonable initial considerations in pediatric patients, persistent or recurrent symptoms should prompt reconsideration of the differential diagnosis. We present a case of a nine-year-old boy with recurrent abdominal pain initially attributed to gastrointestinal causes who was ultimately found to have severe UPJ obstruction with markedly impaired renal function requiring nephrectomy. This case highlights the importance of thoughtful evaluation and early imaging in children with unexplained recurrent and episodic abdominal pain.

## Introduction

Dietl syndrome, often referred to as Dietl crisis during acute presentation, is an uncommon cause of intermittent ureteropelvic junction (UPJ) obstruction characterized by episodic abdominal or flank pain with nausea and vomiting [1-2]. Although UPJ obstruction is a well-recognized congenital anomaly, Dietl syndrome is frequently overlooked in children due to its intermittent presentation and nonspecific symptoms, which often mimic gastrointestinal or functional causes of abdominal pain [1-4]. As a result, diagnostic delays exceeding 12 months have been reported, increasing the risk of irreversible injury secondary to recurrent obstruction, progressive hydronephrosis, and loss of renal function [1].

## Case presentation

A nine-year-old boy presented to a university-affiliated family medicine clinic with complaints of abdominal pain. The patient’s mother accompanied him and assisted with the history. The discomfort had persisted for two days and was generalized, with increased severity in the left lower abdomen. The pain was 5 out of 10 on a numerical scale and did not radiate. There was associated constipation, and the patient’s last bowel movement was five days before presentation. Nausea was also present, but no vomiting had occurred. No home interventions had alleviated the pain. Over-the-counter laxatives and prune juice were administered for constipation, with no improvement. The patient reported continued flatus but no bowel movements. The patient’s oral intake was preserved, and his urine output was consistent. He did not endorse dysuria, hesitancy, urgency, or urinary frequency.

On physical examination, his vital signs were recorded as blood pressure, 125/86 mmHg; heart rate, 80 beats per minute; respiratory rate, 20 breaths per minute; temperature, 97.8 degrees Fahrenheit (36.5 degrees Celsius); and oxygen saturation, 100% on peripheral pulse oximetry. The patient weighed 73.6 pounds (33.4 kg) and was 57 inches (144.8 cm) tall, placing him in the 69th and 90th percentiles, respectively. The documented blood pressure of 125/86 mmHg corresponded to the 98th percentile for age, sex, and height and met the criteria for stage 1 pediatric hypertension. However, it was not flagged as elevated by the electronic health record system. Mild left lower quadrant abdominal tenderness to palpation was present; however, his examination was otherwise normal. No abdominal distention or abnormal bowel sounds on auscultation were observed. The patient appeared well hydrated with normal capillary refill and moist mucous membranes. Cardiopulmonary, neurologic, skin, extremity, and musculoskeletal examinations were within normal limits.

The patient was born at term via normal spontaneous vaginal delivery and had a normal developmental progression. He had a personal history of intermittent constipation and unilateral sensorineural hearing loss, and no surgical procedures. His family history was only positive for his mother’s pulmonary artery stenosis. The child lived with his mother and father, and there was no exposure to second-hand smoke. Although an allergy to hydrocodone was reported, his reaction to that drug was not specified. He took no other prescription or over-the-counter medications.

The patient was diagnosed with acute constipation, and polyethylene glycol (MiraLAX) 17 g per day was prescribed along with lifestyle modification, including increased activity, fluid intake, and dietary fiber. Follow-up was advised for five days later, and abdominal imaging was proposed if symptoms persisted.

**Figure 1:**
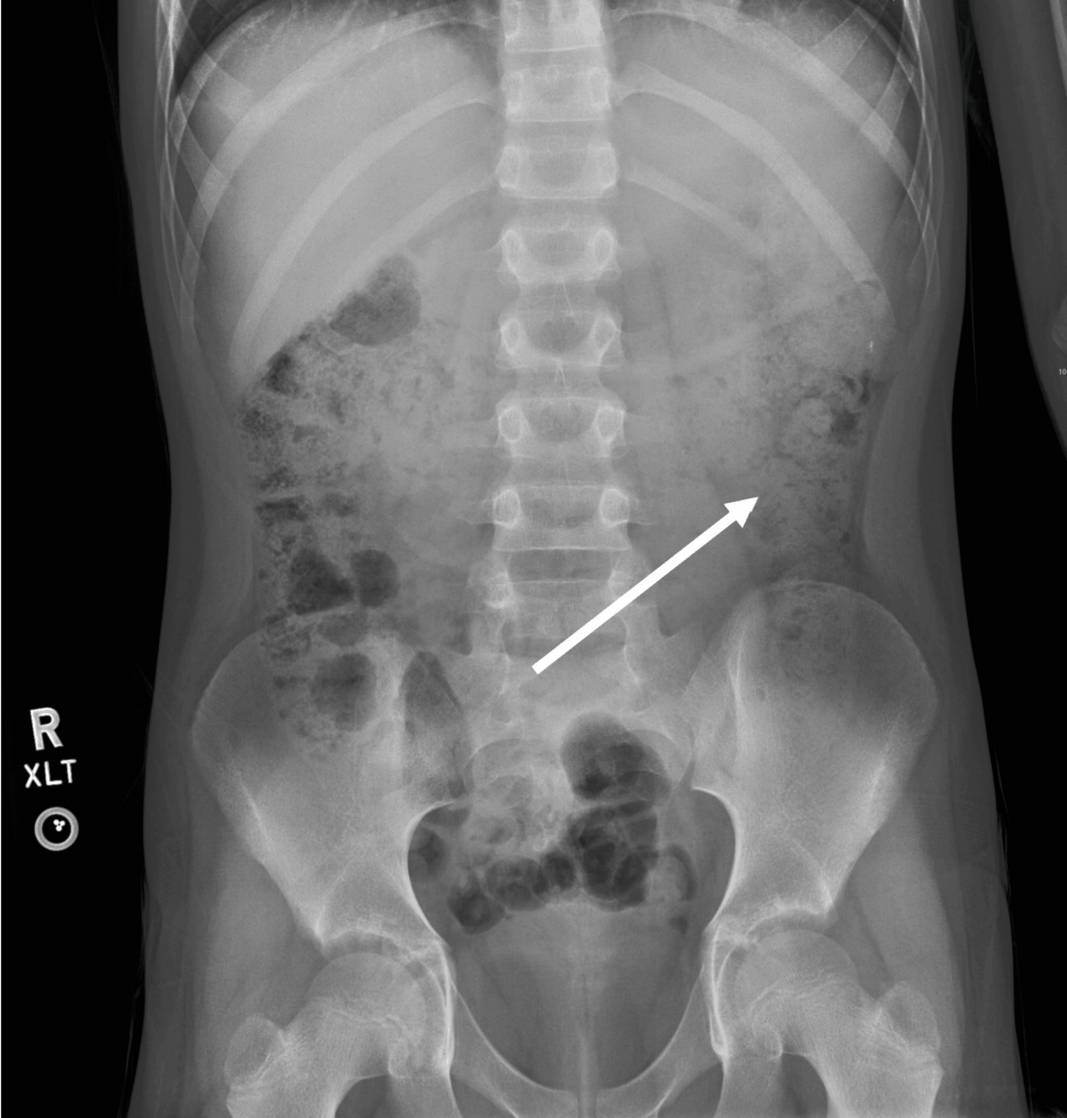
Abdominal KUB radiography demonstrating increased stool volume throughout the colon KUB: kidney-ureter-bladder The arrow points to increased stool burden in the descending colon.

The patient did not return at the scheduled follow-up visit but continued to have intermittent abdominal symptoms over the next 10 months. Several separate encounters occurred during this time, with the patient being diagnosed with varying pathologies, including viral gastroenteritis and gastroesophageal reflux disease (GERD), and being prescribed ondansetron, lactulose, and famotidine. Six months following his initial complaint, he presented with recurrent abdominal pain and constipation, and abdominal kidney-ureter-bladder (KUB) radiography was conducted, which demonstrated an increased volume of stool throughout the colon without obstruction or visible rectal fecal impaction (Figure 1).

Three months following this imaging, he presented again with similar episodic abdominal pain complaints. Significant school absenteeism resulted from his recurring symptoms. His mother reported that his constipation had resolved with the administration of polyethylene glycol. At this time, further investigation was undertaken with laboratory analysis and abdominal ultrasonography. The patient was also referred to gastroenterology with a diagnosis of functional abdominal pain. A clean-catch urinalysis, non-fasting basic metabolic panel (BMP), and complete blood count (CBC) were collected and were largely normal, except for some mild hyponatremia (Tables 1-3).

**Table 1:** Clean-catch urinalysis

Test	Patient’s value	Reference range
Color	Yellow	Yellow
Clarity	Clear	Clear
Specific gravity	1.015	1.001-1.030
pH	5.5	5.0-8.5
Urobilinogen	1.0 EU/dL	0.2-1.0 EU/dL
Glucose	Negative	Negative
Bilirubin	Negative	Negative
Ketones	Negative	Negative
Blood	Negative	Negative
Protein	Negative	Negative
Nitrite	Negative	Negative
Leukocyte esterase	Negative	Negative

**Table 2:** Basic metabolic panel (BMP)

Test	Patient’s value	Reference range
Sodium	134 mmol/L	136-145 mmol/L
Potassium	4.4 mmol/L	3.5-5.1 mmol/L
Chloride	103 mmol/L	98-107 mmol/L
Carbon dioxide (CO_2_)	17 mmol/L	21-32 mmol/L
Anion gap	14	5-15
Glucose	113 mg/dL	70-120 mg/dL
Blood urea nitrogen (BUN)	8 mg/dL	6-19 mg/dL
Creatinine	0.70 mg/dL	0.40-0.70 mg/dL
Calcium	10.0 mg/dL	8.4-10.7 mg/dL
Osmolality	277 mOsm/kg	282-300 mOsm/kg

**Table 3:** Complete blood count (CBC)

Test	Patient’s value	Reference range
White blood cells (WBC)	9.6 × 10^3^/µL	4.5-13.5 × 10^3^/µL
Red blood cells (RBC)	5.32 × 10^6^/µL	4.30-5.90 × 10^6^/µL
Hemoglobin	15.0 g/dL	11.5-15.5 g/dL
Hematocrit	43.6%	35.0-45.0%
Mean corpuscular volume (MCV)	82.0 fL	75.0-87.0 fL
Mean corpuscular hemoglobin (MCH)	28.2 pg	26.0-34.0 pg
Mean corpuscular hemoglobin concentration (MCHC)	34.4 g/dL	31.0-37.0 g/dL
Platelets	423 × 10^3^/µL	140-450 × 10^3^/µL
Red cell distribution width (RDW)	12.5%	11.5-14.5%
Lymphocyte percentage	15.8	20.0-50.0
Mid-sized cell percentage	4.9	2.0-15.0
Granulocyte percentage	79.3	30.0-70.0

A C-reactive protein (CRP) level of < 0.30 mg/dL (reference range, 0.0-0.5 mg/dL) and an erythrocyte sedimentation rate (ESR) of 5 mm/h (reference range, 0-10 mm/h) were within normal limits. A tissue transglutaminase (tTG) antibody, IgA, was negative for celiac disease. Urine culture showed no bacterial growth.

**Figure 2:**
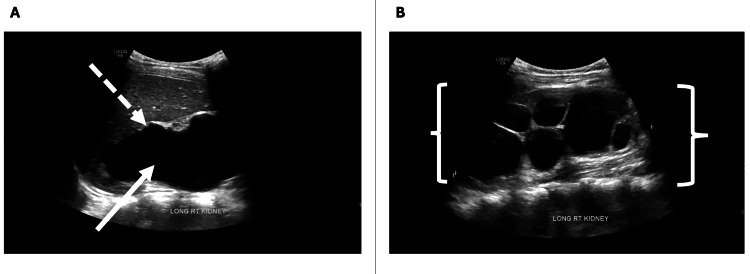
Renal ultrasonography demonstrating severe hydronephrosis of the right kidney A. Longitudinal view showing marked dilation of the renal pelvis and calyces (solid arrow) and cortical thinning (dashed arrow) B. Longitudinal view illustrating continuous dilation of the major and minor calyces producing the classic "bear claw" configuration (brackets)

Abdominal ultrasonography revealed severe right-sided hydronephrosis, most likely secondary to a UPJ obstruction (Figure 2).

A large left kidney was also visualized, suggestive of compensatory hypertrophy. 

**Figure 3:**
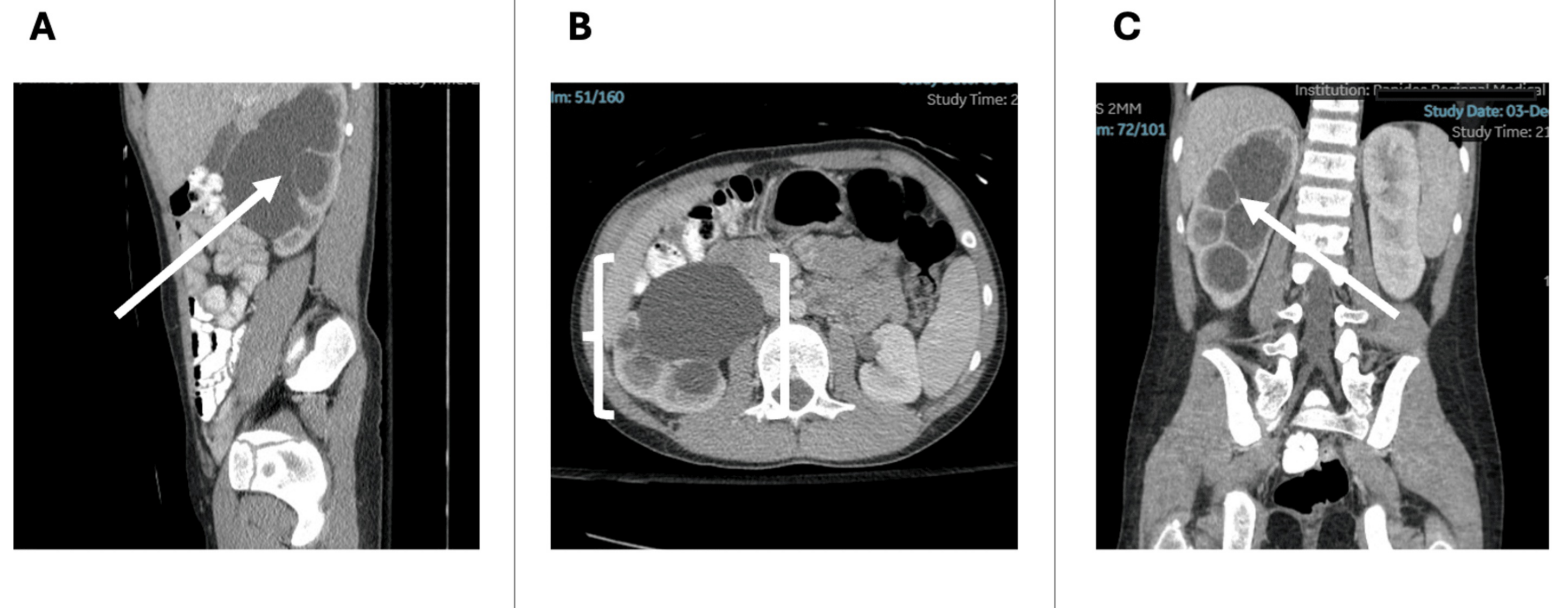
Contrast-enhanced CT of the abdomen and pelvis demonstrating severe right-sided hydronephrosis A. Sagittal view showing marked pelvocaliectasis of the right kidney (solid arrow) B. Axial view demonstrating dilation of the renal pelvis and calyces with classic "bear claw" configuration (brackets) C. Coronal view illustrating extensive calyceal dilation and distortion of the collecting system (solid arrow) CT: computed tomography

An urgent consultation with a pediatric urologist was conducted, and a transfer to a tertiary pediatric referral center for evaluation and advanced imaging was arranged. A computed tomography (CT) abdomen and pelvis with intravenous contrast was ordered and revealed severe right-sided pelvocaliectasis, without a dilated ureter, suggesting a developmental UPJ obstruction (Figure 3).

A confirmatory functional assessment with Technetium-99m mercaptoacetyltriglycine (MAG3) diuretic renography was obtained. The study demonstrated a markedly prolonged drainage half-time (T½) of 554.8 min (reference range < 20 min) and reduced right renal differential function of 21.4% (reference range 45-55%), consistent with long-standing UPJ obstruction. Given the markedly prolonged drainage time, reduced differential renal function, severe hydronephrosis with cortical thinning, and overall clinical context suggesting longstanding obstruction, pediatric urology determined that the likelihood of meaningful functional recovery was low. Although pyeloplasty was considered, the combination of functional impairment, structural changes, and chronicity of disease supported the decision to proceed with nephrectomy rather than reconstructive intervention. The procedure was completed without complication.

**Figure 4:**
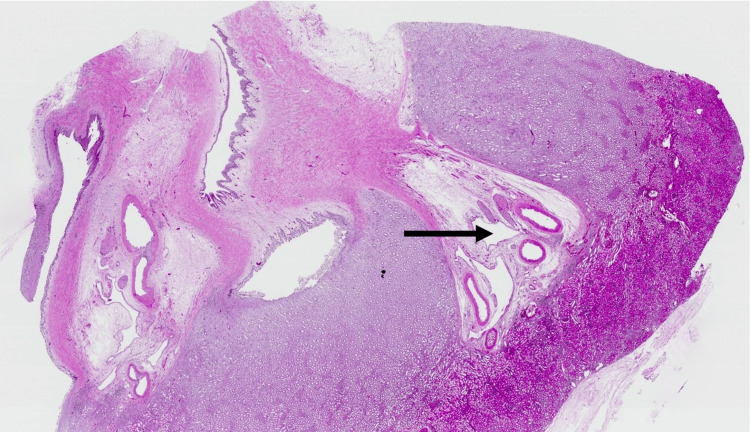
Hematoxylin and eosin-stained section of kidney at 40× original magnification Dilated renal calyx (arrow) consistent with hydronephrosis

Histopathologic examination of the resected kidney confirmed chronic hydronephrosis with marked calyceal dilation, cortical thinning, and interstitial fibrosis, consistent with prolonged obstructive uropathy (Figure 4).

Postoperatively, the patient experienced complete resolution of abdominal pain and related symptoms and returned to school and normal daily activities. Follow-up renal ultrasonography 11 months later demonstrated the absence of the right kidney, consistent with previous nephrectomy. The left kidney measured 14.2 cm and demonstrated normal parenchymal echogenicity with compensatory hypertrophy. The urinary bladder was grossly unremarkable. These findings were interpreted as adaptive changes following unilateral nephrectomy. The patient remained clinically asymptomatic with preserved renal function on surveillance and required no further intervention. A simplified chronological representation of the patient's clinical course is summarized (Table 4).

**Table 4:** Clinical timeline from initial presentation to diagnosis of Dietl syndrome KUB: kidney-ureter-bladder, CT: computed tomography, CBC: complete blood count, BMP: basic metabolic panel, GERD: gastroesophageal reflux disease, UPJ: ureteropelvic junction, IV: intravenous, BUN: blood urea nitrogen, MAG3: mercaptoacetyltriglycine

Encounter	Location	Time	Diagnosis	Lab/imaging evaluation	Intervention
1	University-affiliated family medicine clinic	Day 1	Constipation	None	Polyethylene glycol and lifestyle modification
2	University-affiliated family medicine clinic	Day 176	Viral gastroenteritis	None	Ondansetron
3	University-affiliated family medicine clinic	Day 191	Constipation	KUB	Refill polyethylene glycol
4	University-affiliated family medicine clinic	Day 226	Constipation	None	Lactulose
5	University-affiliated family medicine clinic	Day 267	GERD	None	Famotidine
6	University-affiliated family medicine clinic	Day 283	Functional abdominal pain	CBC, BMP, urinalysis/abdominal ultrasound	Referral to pediatric gastroenterology
7	University-affiliated family medicine clinic	Day 287	Right-sided hydronephrosis	CT abdomen and pelvis with IV contrast	Urgent referral to pediatric urology
8	Tertiary pediatric referral center	Day 310	Dietl syndrome (intermittent UPJ obstruction)	MAG3 renal study	Right nephrectomy
9	University-affiliated family medicine clinic	Day 407	Well child	BUN and creatinine	Continued surveillance

**Figure 5:**
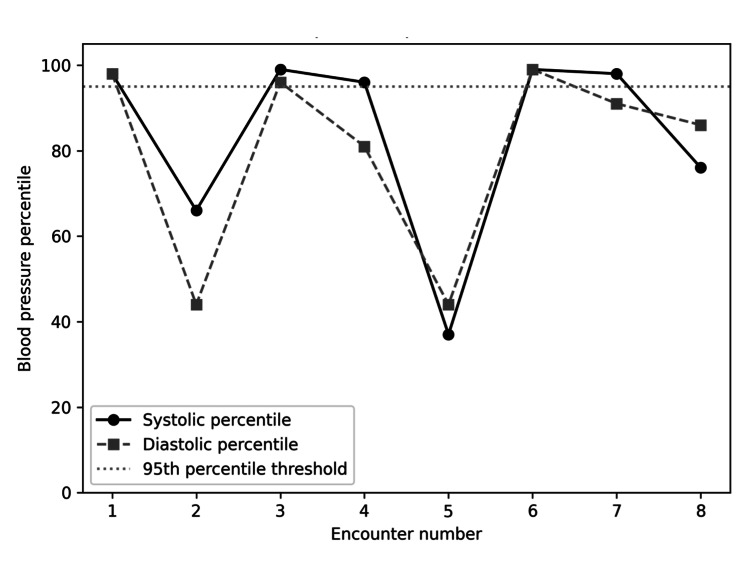
Serial blood pressure percentile trends

Upon review of the patient’s clinical course, it was retrospectively identified that the patient’s measured blood pressure values were elevated during several encounters, a finding that was not initially recognized. Several readings during separate visits to the family medicine clinic were above the 95th percentile for age, sex, and height, meeting the criteria for pediatric hypertension (Table 5 and Figure 5).

**Table 5:** Blood pressure measurements, percentiles, and hypertension classification during clinical course

Blood pressure (mm/Hg)	Percentile (systolic/diastolic)	Hypertension category
125/86	98/98	Stage 1
108/60	66/44	Normal
128/82	99/96	Stage 1
122/72	96/81	Stage 1
100/60	37/44	Normal
139/94	99/99	Stage 2
127/78	98/91	Stage 1
113/75	76/86	Normal

## Discussion

Dietl syndrome is a clinical manifestation of intermittent UPJ obstruction characterized by episodic abdominal or flank pain accompanied by nausea and vomiting, often referred to as Dietl crisis during acute presentation. First described by Josef Dietl in 1864, the syndrome results from transient obstruction of urinary flow at the UPJ, leading to acute renal pelvic distension during periods of increased diuresis [1-5]. Exacerbations of Dietl syndrome often occur following episodes of increased oral intake and excessive hydration, as well as during periods of diuresis, such as after caffeine or alcohol intake. Episodes may last for minutes to hours, followed by long periods of symptom dormancy. Associated presentations may include fatigue and decreased appetite, which can result in weight loss and growth faltering. UPJ obstruction is a well-recognized congenital anomaly; however, Dietl syndrome remains underdiagnosed in pediatric populations secondary to its intermittent presentation and frequent misattribution to gastrointestinal or functional disorders [3-4,6]. Importantly, the initial attribution of symptoms to gastrointestinal causes in this case was not inherently inappropriate given the patient’s presentation; however, the persistence and recurrence of symptoms without sustained response to therapy warranted earlier diagnostic reconsideration.

UPJ obstruction is the most common congenital cause of hydronephrosis in children, with an estimated incidence of approximately 1 in 1,000-2,000 live births worldwide [1-3]. However, only a subset of affected patients develops intermittent obstruction consistent with Dietl syndrome. Previous studies suggest that approximately 11-15% of pediatric patients with UPJ obstruction present with acute, episodic abdominal pain consistent with Dietl crisis [1-3]. The condition most commonly affects school-aged children, with a mean age of presentation around 10-12 years, and demonstrates a clear male predominance, with reported male-to-female ratios ranging from 2:1 to 3:1 [1-3]. No consistent racial or ethnic predilection has been identified.

**Figure 6:**
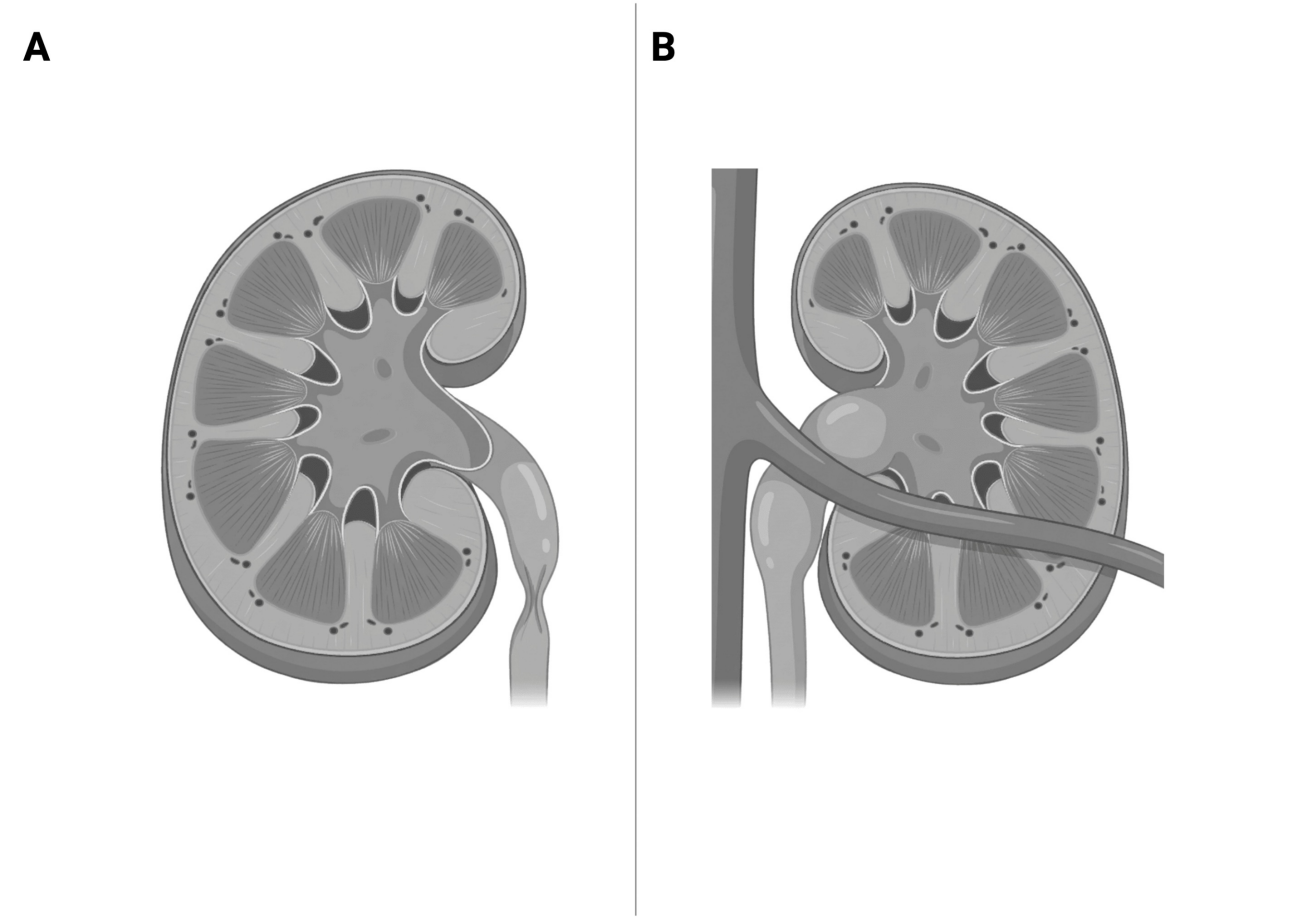
Schematic illustration of intrinsic and extrinsic UPJ A. Intrinsic UPJ obstruction caused by congenital narrowing or abnormal muscular development of the ureteropelvic junction, resulting in impaired drainage of urine from the renal pelvis into the ureter. B. Extrinsic UPJ obstruction caused by compression from an aberrant lower pole crossing vessel, resulting in intermittent obstruction of urinary flow. UPJ: ureteropelvic junction Created with BioRender.com by Pippin M. No artificial intelligence tools were used to create this image (https://BioRender.com/flioxef)

The pathophysiology of Dietl syndrome involves intermittent obstruction at the UPJ caused by intrinsic or extrinsic mechanisms [2-4,7-9]. Intrinsic causes include congenital narrowing, fibrosis, or abnormal smooth muscle development of the UPJ, whereas extrinsic causes most commonly involve compression from aberrant lower pole crossing vessels (Figure 6) [1-4].

Dynamic factors, such as position-dependent kinking and abnormal peristalsis, further contribute to transient obstruction, particularly during periods of increased urine output [1-4]. These episodes result in acute renal pelvic distension and activation of renal capsular stretch receptors, producing visceral pain that is frequently perceived as abdominal rather than flank pain in children [1-4].

When left untreated, Dietl syndrome can result in progressive renal injury due to repeated episodes of transient hydronephrosis and renal ischemia [1-4,6]. Recurrent ischemic insults induce tubular atrophy, interstitial fibrosis, and glomerulosclerosis, leading to irreversible parenchymal loss and reduced differential renal function [1-3]. Long-term consequences include cortical thinning, increased susceptibility to urinary tract infections, secondary hypertension, and, in severe or prolonged cases, chronic kidney disease [5,6]. Importantly, delayed surgical intervention has been associated with diminished recovery of renal function compared with early correction, underscoring the importance of timely diagnosis and intervention [5].

Although hypertension is not a classically described hallmark of Dietl syndrome, recurrent UPJ obstruction may provide a physiologic mechanism for secondary hypertension through activation of the renin-angiotensin-aldosterone system in response to increased intrarenal pressure and reduced renal perfusion [1-4]. Renin release from the juxtaglomerular apparatus promotes angiotensin II-mediated vasoconstriction and aldosterone-mediated sodium and water retention, ultimately resulting in systemic hypertension [1-4]. In our patient, several blood pressure values met criteria for pediatric hypertension, including one reading representing stage 2 hypertension, none of which were identified as abnormal during clinical encounters [10]. This missed opportunity is consistent with current literature suggesting pediatric hypertension often goes underdiagnosed due to complex age, sex, and height-based percentile calculations and electronic health record software inconsistently flagging abnormal values [10-11]. However, elevated blood pressure readings in pediatric patients may also reflect transient factors such as pain, stress, measurement variability, or improper cuff sizing, and therefore should be interpreted cautiously in isolation [10-11]. Still, failure to recognize elevated blood pressure may delay consideration of renal pathology in children presenting with nonspecific abdominal symptoms, as occurred in this case [10-11].

Several additional factors contribute to the delayed diagnosis of Dietl syndrome, including intermittent symptom resolution, absence of urinary complaints, normal laboratory findings, and early diagnostic anchoring on functional gastrointestinal conditions such as constipation, abdominal migraine, or psychosomatic pain [1-2]. As illustrated in the present case, initial reliance on abdominal radiography and the identification of fecal loading contributed to a presumptive diagnosis of constipation, delaying renal evaluation for several months. However, fecal loading on abdominal radiography is a nonspecific finding that does not reliably correlate with symptom severity or confirm constipation as the primary etiology of pain [1-3]. In pediatric patients, stool burden may coexist with other pathologies, and overreliance on this finding may contribute to diagnostic anchoring [1-3]. This bias pattern is consistent with previous reports describing diagnostic delays exceeding 12 months in pediatric patients with Dietl syndrome [1-3,9,12-13].

In retrospect, several clinical features in this case were not fully integrated into a unifying diagnostic framework and may have served as early indicators of a non-gastrointestinal etiology. These included the recurrent and episodic nature of the pain over an extended period, accumulation of healthcare encounters without a durable diagnosis, significant school absenteeism, limited response to gastrointestinal-directed therapies, and the absence of sustained clinical improvement.

Physical examination findings are often unremarkable in Dietl syndrome, particularly between symptomatic episodes [1-4,14]. During acute attacks, patients may demonstrate upper abdominal or flank tenderness without peritoneal signs [2-4]. Fever and systemic toxicity are typically absent unless secondary infection has occurred [2-4]. Laboratory studies, including serum creatinine, blood urea nitrogen, electrolytes, and urinalysis, are usually normal unless advanced or bilateral disease is present [1-5].

Imaging plays a central role in the diagnosis of Dietl syndrome [1-5,7]. Renal ultrasonography, including point-of-care ultrasonography (POCUS), is the preferred initial modality and may demonstrate hydronephrosis or pelvicalyceal dilation, although findings can be intermittent and absent between episodes [1-2,4-5]. In the context of vague, undifferentiated abdominal complaints, a complete abdominal ultrasound is more likely to be ordered by providers and can appropriately identify renal pathology, although a targeted renal ultrasound is recommended specifically when Dietl syndrome is suspected [1-2]. Doppler ultrasonography may be used to assess renal perfusion [1-3]. Functional imaging with Technetium-99m MAG3 diuretic renography remains the gold standard for confirming obstruction and assessing split renal function [1-4]. A prolonged half-time to drainage (T½ >20 min) supports the diagnosis of obstruction and guides management decisions [1-2]. In the present case, the markedly prolonged drainage time (T½ = 554.8 min) and reduced differential renal function (21.4%), together with the overall clinical picture, indicated a longstanding obstruction with limited potential for renal salvage. Such a significantly prolonged T½ value can occur when washout is minimal, reflecting near-absent drainage rather than a precise measurable T½ [1-2].

Dietl syndrome is a noninfectious process, and urine cultures are typically negative [1-3]. Histopathologic evaluation is not required for diagnosis but may be obtained following nephrectomy [1-3]. Characteristic findings include chronic hydronephrosis, tubular atrophy, interstitial fibrosis, cortical thinning, and glomerulosclerosis, all of which reflect irreversible renal injury resulting from prolonged obstruction [1-3].

Management depends on symptom severity and residual renal function. In patients with preserved renal function, pyeloplasty, performed via open, laparoscopic, or robotic approaches, is the preferred intervention and achieves success rates exceeding 90% [1,3-4]. Nephrectomy is indicated when renal function is severely impaired, generally defined as split function of 10-20% or less, or when longstanding obstruction suggests minimal potential for functional recovery [1-2]. Although pyeloplasty was considered in this case, and the split function value did not quite reach the 20% threshold for severe disease, the degree of functional impairment and chronicity of obstruction favored nephrectomy as directed by the pediatric urologist. Following surgery, the patient experienced complete resolution of symptoms, reinforcing the causal relationship between the obstructed kidney and his abdominal complaints.

This report has several limitations. As a single case report, conclusions regarding prevalence and optimal diagnostic strategies cannot be generalized. Additionally, the patient’s symptoms were intermittent and often resolved between clinical encounters, which likely contributed to diagnostic anchoring on gastrointestinal etiologies and delayed renal imaging. Finally, retrospective review of clinical records may incompletely capture all clinical decision-making factors present during initial encounters.

## Conclusions

Dietl syndrome is an uncommon but important manifestation of long-standing, recurring UPJ obstruction, which may lead to chronic kidney injury and, in severe cases, nephrectomy. Dietl syndrome should be considered in children with recurrent unexplained abdominal pain, particularly when symptoms are episodic, associated with nausea or vomiting, refractory to initial management, or when clinical features are not fully explained by common gastrointestinal diagnoses. Provider awareness and a high index of suspicion, along with prompt ultrasonography, may facilitate early recognition and reduce diagnostic delays, preventing long-term complications.
